# Synthesis of vinyl ester resin-carrying PVDF green nanofibers for self-healing applications

**DOI:** 10.1038/s41598-020-78706-3

**Published:** 2021-01-13

**Authors:** C. Naga Kumar, M. N. Prabhakar, Jung-il Song

**Affiliations:** Department of Mechanical Engineering, Changwon National University, Changwon, 51140 Korea

**Keywords:** Engineering, Materials science

## Abstract

Self-healing on the engineering applications is smart, decisive research for prolonging the life span of the materials and the innovations have been mounting still smarter. Connecting to advancements in self-healing carriers, in altering the chemical structure by optimizing the brittleness for self-healing performance and introducing the bio-degradability, for the first time TPS was blended to PVDF for the synthesis of nanofibers, as carriers of a vinyl ester (VE) resin (medication), by the coaxial electrospinning technique. TPS was mechanically mixed with PVDF base polymer and optimized the TPS content (10 wt%) based on mechanical performance. The novel nanofibers were characterized via field emission scanning electron microscopy (FESEM), Fourier-transform infrared spectroscopy, X-ray diffraction, thermal, moisture analysis, and a mechanical line with FESEM and energy-dispersive X-ray analysis studied the self-healing. The TPS/PVDF fibers having hydrogen bonding and increased the crystallinity (40.57 → 44.12%) and the diameter (115 → 184 nm) along with the surface roughness of the fibers with increasing the TPS content. Microanalysis presented the flow-out of the VE resin at the scratched parts in the pierced fibers; interestingly, after some time, the etched part was cured automatically by the curing of the spread resin. Mechanical stretching of the nanofibers in the tensile tests up in the plastic region showed a decrement in the elasticity (TPS/PVDF fibers) and an increment in the brittle nature (cured VE resin) with the increase in Young’s modulus at each stretching, clearly elucidating the healing performance.

## Introduction

In the present decade, researchers have been mostly interested in using nanofibers as smart carriers for self-healing applications, e.g., curing microcracks, corrosion protection coatings for metals, and wound healing^1^. The usage of self-healing nanofibers can extend the usability and life span of materials/composites. Nanofibers have excellent properties, such as a large surface area to volume ratio, a high degree of flexibility, and good mechanical properties^2^. Electrospinning has become a well-known technique to investigators for the preparation of nanofibers owing to its easy operation under optimized conditions compared to all the methods^3^. In this technique, an electrostatic force is applied to eject a polymer solution jet from the tip of a droplet to form nanofibers. In the early years of the twenty-first century, technological developments led researchers to modify the electrospinning method to prepare tube-like structure (core–shell) nanofibers with distinctive features by installing a particular type of nozzle, called a coaxial nozzle^4–6^. In the self-healing field, this innovation tremendously promoted the use of self-healing carriers as regenerative medicine in tissue engineering^7^, releasing healing resin in mechanical engineering^8^, etc.

The utilization of nanofibers in self-healing applications is particularly attractive compared to the existing self-healing carriers, e.g., microspheres and vascular networks, owing to the limitations of uniform distribution and uneven surfaces of the latter in real products. Importantly, in the above two cases, more carriers are required for both the healing resins along with a curing agent, which increases the density of the corresponding composites and makes the healing mechanism complex^9–11^. In fact, microsphere reinforcement in composites by the vacuum-assisted resin transfer molding (VARTM) process is highly complex. Moreover, the hardener microspheres should be distributed on the resin microspheres, particularly at the cracked parts, which is highly impossible in composites for achieving an effective self-healing process^12^. Recently Maryam et al., studied self-healing behavior by developing silane coupled cellulose-functionalized halloysite nanotube/epoxy nanocomposites^13^.

Several thermoplastic polymers have been used to synthesize nanofibers by the electro-spinning method such as polyacrylamide (PAM), polyvinyl alcohol (PVA), polyvinyledene fluoride (PVDF), etc.^14,15^. PVDF is a well-known polymer mostly used to produce nanofibers owing to its excellent mechanical, thermal, and electrical properties^16^. It has a semi-crystalline structure and is non-biodegradable and hydrophobic in nature. Numerous researchers have studied the development of the electrical behavior of PVDF nanofibers of chemical modifications and reinforcement of various additives^17,18^; in addition, a few of them have focused on their mechanical properties and hybridization. For example, (Dai et al.) self-healing internal electric field is proposed and successfully endowed to a designed helical structural composite microfiber polyvinylidene fluoride/g-C3N4) based on the bio inspired simple harmonic vibration^19^. Aatif et al. confirmed an improvement in the mechanical properties of the β-phase fraction of PVDF nanofibers when bentonite nano clay particles were introduced into the PVDF dope solution for electrospinning^20^. Pu et al. observed an increment in Young’s moduli, tensile strength, and thermal stabilities of PVDF nanofibers when combined with ZnO nanowires^21^. Seyyed et al. proved that the mechanical properties of PVDF nanofibers varied with the increase in nano silica content and stated that with higher SiO_2_ content, PVDF nanofibers with better tensile strength, more toughness, and smaller elongation to break were obtained^22^.

In fact, PVDF nanofiber-reinforced composites are suitable to be used as self-healing carriers. Mahanty et al. fabricated PVDF/MWCNT composite electrospun nanofibers followed by piezoelectric pressure sensor directly as self-powered electronic skin (e-skin)^23^. Wook et al. used PVDF as shell material in self-healing nanofibers; the authors encapsulated two components (a resin monomer and a hardener) separately in the cores of PVDF nano and microfibers formed by solution blowing using compressed air. The self-healing properties of such nano and microfiber mats were explored using blister tests along with periodic bending fatigue tests^24^. PVA/PVDF core–shell nanofibers were synthesized by Yuhua et al., and showed that MBT-loaded coaxial nanofibers decreased the corrosion activity of coatings^25^. Choolaei et al. confirmed the improvement in the crystallinity of PVDF-HPF nanocomposites by adding GO nanoparticles through a heterogeneous nucleation mechanism^26^.

Indeed the flexible nature of PVDF Nanofibers is not well support the significant self-healing performance due to damage issues under loadings^27^. Optimizing brittleness for achieving adequate healing performance, especially in connection to micro-cracks in fiber-reinforced self-healing composites is a critical task until no researchers focused. Hence, the current research attempts to practically realize the above-mentioned hypothesis advancements and problems in PVDF, for the first time by chemical blending low-cost starch in the form of a thermoplastic polymer for optimizing the brittle nature towards effective self-healing performance as well as introducing biodegradability. A coaxial electrospinning instrument (Model: NS1 NanoSpinner Electrospinning equipment, INOVENSO, Korea) was used to produce core–shell nanofibers with uni-diameters. The mechanical properties were studied for various weight percentages of thermoplastic cornstarch (TPS) (5, 10, 15, and 20 wt%). As expected, the crystallinity and the modulus of the fibers increased with the increase in the TPS weight percentage in PVDF, which, in turn, increased their diameter, as confirmed by X-ray diffraction (XRD), tensile tests, and field emission scanning electron microscopy (FESEM). 10 wt% of TPS blended PVDF polymer was chosen to use for further study, even though the higher percentage of TPS (15 and 20 wt%) showing higher properties such as mechanical strength, surface roughness, degree of crystallinity, water absorption etc., due to mainly on the structure formation of the uniform diameter of nanofiber without any obstructions. The self-healing roles of the fabricated nanofibers were investigated by periodic stretching by tensile loading up in their elastic zone. The variation in the modulus and morphology significantly affected the healing performance of the TPS/PVDF core–shell nanofibers.

## Materials and methodology

### Materials

PVDF and glycerol (ACS reagent ≥ 99.5%) were purchased from Sigma-Aldrich, USA, and dimethylformamide (DMF) (99.0%) was purchased from Samchun Chemicals, South Korea. Corn starch (72% amylopectin and 28% amylose) was received from Samyang Corporation Ltd., South Korea. Vinyl ester (VE) (viscosity = 150 cps and specific gravity = 1.03), methyl ethyl ketone peroxide (MEKP) (catalyst), and cobalt naphthalate (accelerator) were procured from CCP Composites, Korea.

### Preparation of self-healing nanofibers

**Figure 1:**
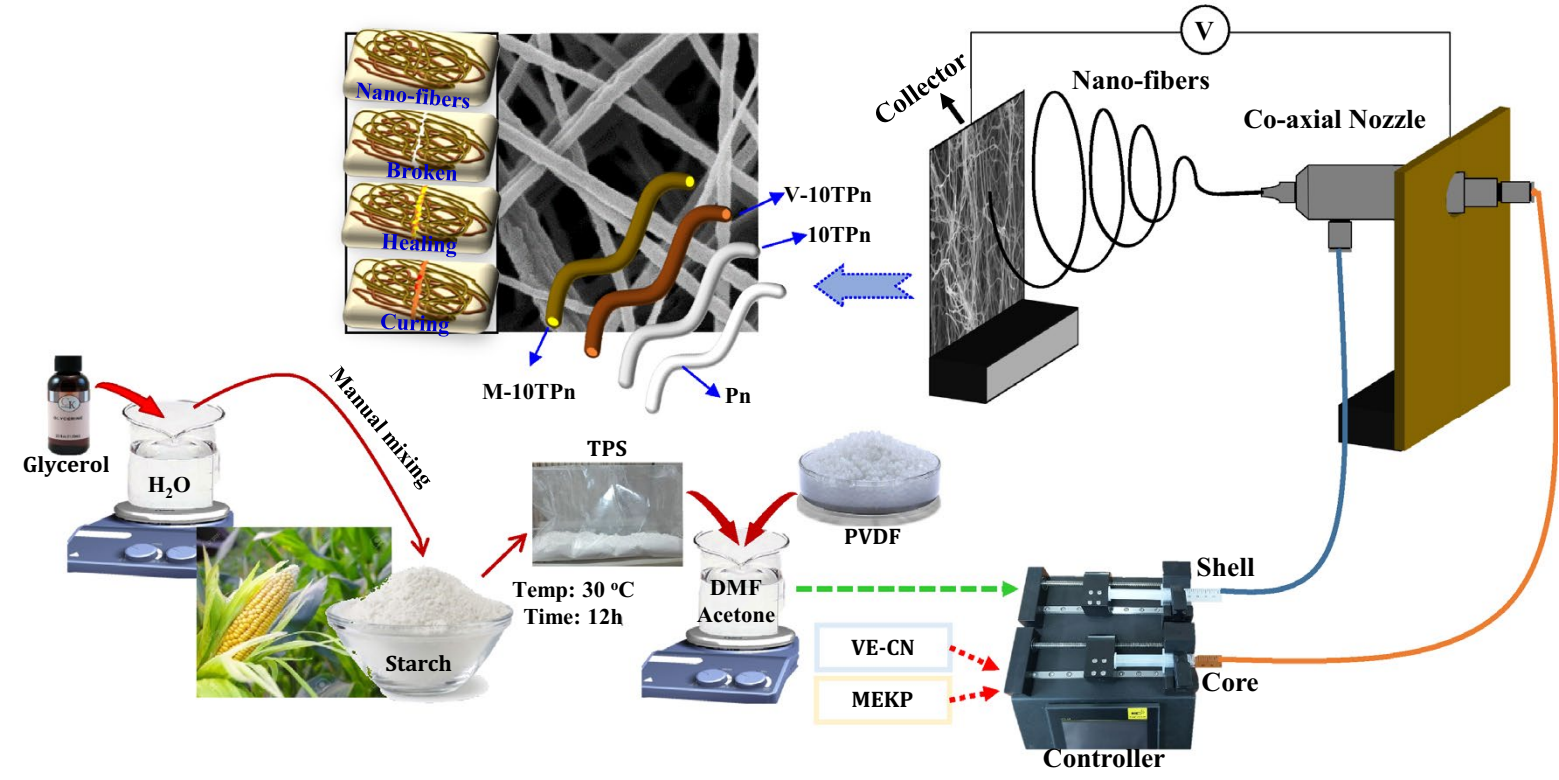
Schematic representation of electrospinning process producing TPS/PVDF core–shell nanofibers.

The fabrication process of TPS/PVDF core–shell nanofibers as shown in Fig. 1, consist of mainly four steps as follows: Step 1: Synthesis of TPS, Step 2: Preparation of TPS/PVDF polymer blend solution, Step 3: Preparation of core (VE–CN) and MEKP solutions and Step 4: Fabrication of TPS/PVDF core–shell nanofibers. The detailed information about the steps is discussed below.

*Step 1: Synthesis of TPS*

TPS was prepared by a simple process in which glycerol and deionized (DI) water were taken in a 3:1 ratio and stirred at a temperature of 80 °C for more than 10 min to form a homogeneous solution. Subsequently, the obtained solution and starch were mixed together in a ratio of 1:2 for producing the required TPS^28^.

*Step 2: Preparation of TPS/PVDF blend solution*

A 1:3 (w/w) acetone/DMF mixture was used as the solvent, to which 18 wt% PVDF was added. The mixture was stirred at 80 °C for 8 h, and the prepared TPS (10 wt%) was then added to the PVDF solution. The stirring was continued for 6 h at 80 °C until a homogeneous TPS/PVDF solution was obtained.

*Step 3: Preparation of core solutions (VE–CN and MEKP)*

The effective curing process of vinyl ester (VE) requires both accelerators (CN) and catalyst (MEKP). Hence, three types of core have to prepare such as VE, CN, and MEKP. However, to minimize the density of the nanofibers only two types of core materials (VE–CN and MEKP) used based on chemical nature. VE–CN solution was prepared by adding the required amount of CN to the VE (100:1) and mixing thoroughly on an overhead stirrer for 10 min at room temperature. MEKP was used directly without further modification.

*Step 4: Synthesizing green PVDF nanofibers*

PVDF and TPS/PVDF nanofibers were synthesized using an electrospinning machine with an electrospinning needle. To synthesize the core–shell nanofibers by coaxial electrospinning, a coaxial nozzle with an inner diameter of 0.8 mm and an outer diameter of 1.6 mm was used, as shown in Fig. 1. VE resin and cobalt naphthalene were mixed in a 100:1 ratio and were used as the core materials such as VE–CN, and MEKP. The flow rate of the TPS/PVDF (shell) solution was maintained at 1 mL/h, and those of the VE–CN resin (core) and MEKP (core) were maintained at 0.08 mL/h each. A nozzle tip and a collector were fixed at a distance of 15 cm, and a current–voltage of 18 kV was supplied to induce the nanofiber formation from the solution. Both the VE–CN and MEKP core–shell nanofibers were electrospun on the same collector successively to entangle and form a self-healing core–shell nanofiber mat. Consequently, in individual nanofibers, the core materials (VE–CN and MEKP) were stored in a liquid state as the core of the core–shell nanofibers, which was surrounded by the TPS/PVDF shell.

### Testing and characterizations

Nanofiber mats of 25 × 15 mm in size were tested using a UTM machine (2.5 ton load, R&B Inc, South Korea) at a cross-head speed of 1 mm/min. Attenuated total reflection (ATR)–Fourier-transform infrared (FTIR) spectra of the fibers were measured by an FTIR spectrometer (FT-IR-6300, JASCO International Co., Ltd., spectrometer, Japan). The FTIR spectra were collected in a range of 4000–400 cm^−1^. XRD patterns were obtained using an X-ray diffractometer (Bruker, D8 Discover). The samples were mounted on the sample holder, and the patterns were recorded by running the instrument at a speed of 2°/min and a 2θ range of 10°–40°. The crystallite sizes of the nanofibers were calculated by Scherrer’s equation ($\mathrm{D }=\mathrm{ K\lambda }/\mathrm{\beta cos\theta }$), and the degree of crystallinity was obtained by the following equation:

$$\text{Degree of crystallinity}= \left(\frac{Area\; of \;crystalline \;peaks}{Area \;of \;crystalline \;peaks+Amorphous \; peaks}\times 100\right)$$

The thermal stability was characterized via thermogravimetric analysis (TGA) (STA 6000, PerkinElmer, UK) within a temperature range of 30–700 °C and at a rate of 20 °C/min under a nitrogen atmosphere. To study the surface morphology, the specimens were examined via FESEM (LYRA3xm, Czech Republic) at an accelerated voltage of 5–30 kV with an energy-dispersive X-ray (EDX) analyzer. All the samples were sputter-coated with gold using an automated fine coater (JEOL JFC-1600). A water absorption test was conducted on the nanofibers by soaking them in water for 24 h. The weights of the specimen before (m_0_) and after (m_1_) the soaking were noted, and the water absorption (Q) was calculated using the formula, $Q=\frac{\mathrm{m}1-\mathrm{m}0}{\mathrm{m}0}\times 100$^29^. Five samples were tested for each type of nanofiber, and all the samples were pressed with tissue paper and dried before weighing.

## Results and discussion

### Analysis of TPS/PVDF nanofibers

#### Microanalysis

**Figure 2:**
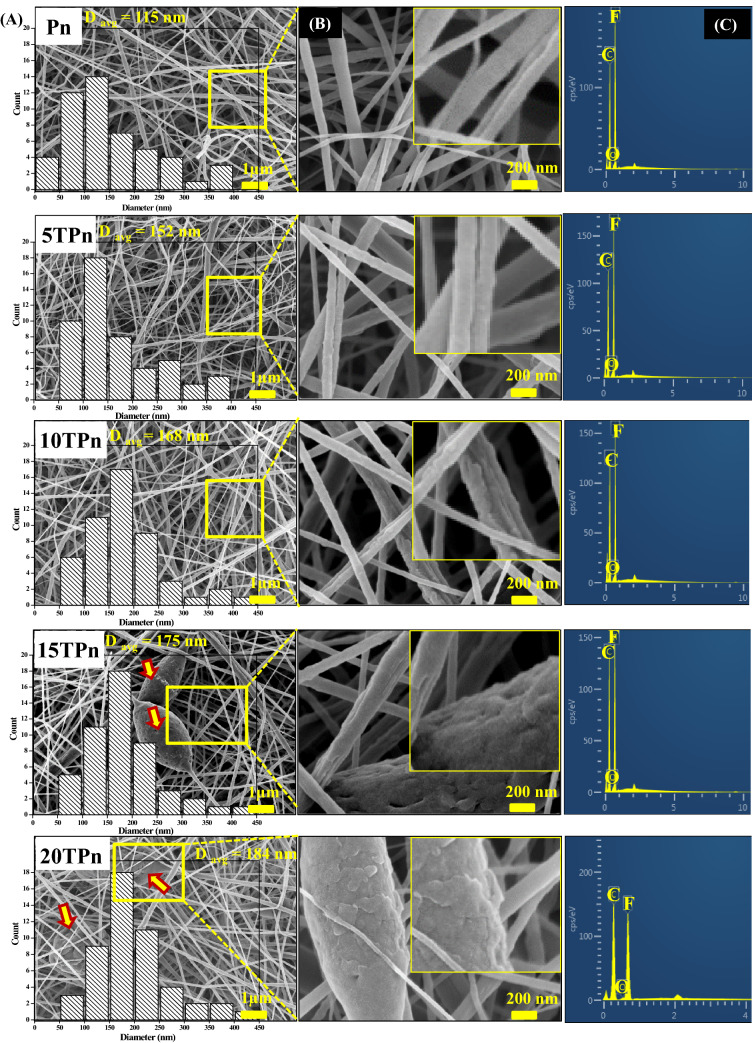
TPS/PVDF nanofibers: (**A**) Diameter distribution histogram and FESEM images at less magnification, (**B**) high magnification FESEM images and (**C**) EDX analysis.

Figure 2A shows the FESEM images of the PVDF nanofibers with different weight percentages of TPS and confirms the presence of nanofibers with accurate nanosized diameters. The diameter distribution histogram presents the diameters of the nanofibers by counting 50 nanofibers using the Nano Measurer software. The average diameter of Pn is approximately 115 nm. As the TPS weight percentage in PVDF increases, the average diameter of the TPS/PVDF nanofibers also increases as follows: Pn (115 nm)  < 5TPn (152 nm)  <  10TPn (168 nm)  < 15TPn (175 nm)  < 20TPn (184 nm)^30^.

The surfaces of the nanofibers tremendous change with the variation in the TPS content, as shown in the second column of the zoomed images in Fig. 2B; it can be seen that the PVDF nanofibers have a smooth surface, which becomes rough as the percentage of TPS increase. This is probably because the introduction of TPS causes a decrease in the conductivity of the electrospinning solution, which assists in increasing both the fiber diameter and the roughness^31,32^.

The roughness of these fiber surfaces suggest that they are brittle, which can be confirmed by the increment in the tensile modulus, as explained later in “Tensile properties” section. The crests and troughs on the surfaces of the fibers also help in the interfacial bonding between the fibers and a resin when they are used for the preparation of composites. Nanofibers with collapsed beads and micrometer-sized droplets of the solution are observed (Fig. 2B) as the TPS content reached 15 wt% and more. This can be attributed to the high viscosity and surface tension of the TPS/PVDF solution, causing the jet to interrupt and withdraw into spherical forms^33^. Because of the presence of more beads and spherical droplets in 15TPn and 20TPn, they cannot be used further for preparing core–shell nanofibers. Thus, 10TPn is considered as the optimal polymer blend based on the morphological observations. The compositions of the elements in the fibers were determined by EDX analysis, and the results are shown in Fig. 2C. The content of fluorine decreases gradually as the TPS content increases, which suggests a reduction in the PVDF percentage in the nanofibers with an increase in TPS.

#### Spectral analysis

**Figure 3:**
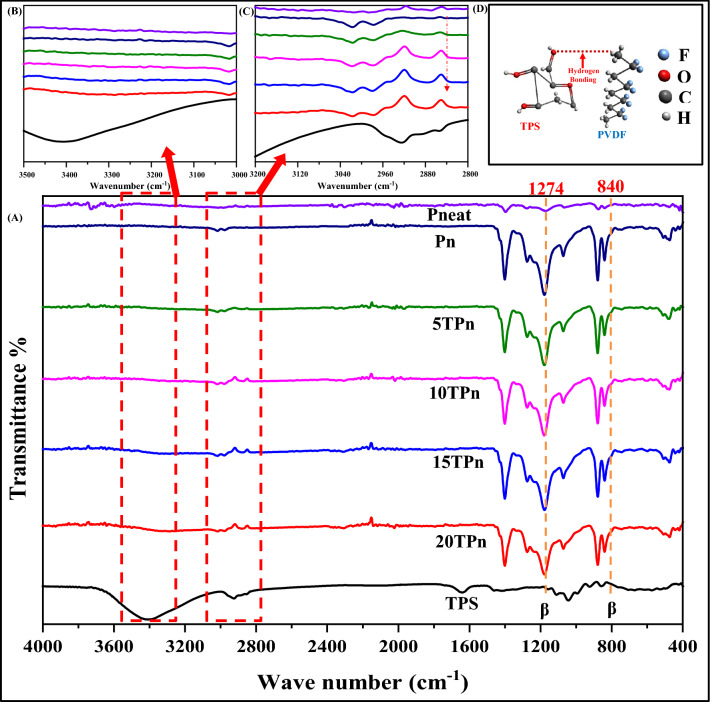
(**A**) FTIR spectra including (**B**,**C**) new characteristic bands of TPS/PVDF nanofibers and (**D**) Molecular interaction between TPS and PVDF.

The interfacial interaction and confirmation of hydrogen bonding were characterized using FTIR. Figure 3A shows all the crystalline phases can be recognized through the vibrational peaks between 1300 and 600 cm^−1^ in the fingerprint region. Accordingly, PVDF may present at least four different well-known crystalline structures: the orthorhombic alpha (α), beta (β), gamma (γ), and the monoclinic delta (δ) phase in terms of its crystallization conditions. However, in PVDF nanofibers, the FTIR spectral doesn’t show any vibrational bands at 976, 796, 764 and 614 cm^−1^ and also at 1232, 833, 812 and 776 cm^−1^ which indicates the absence of α phase and γ phase in which PVDF neat possessed^34^. Thus, the peaks 1401, 1274 and 840 cm^−1^ exclusively represent the characteristics of the β phase.

Observing the FTIR peaks of TPS, the characteristic bands around at 3425, 3270 and 2923 cm^−1^ (CH and OH stretching bands), 1640 cm^−1^ (absorbed water), 1152, 1018 and 1103 cm^−1^ (CC and CO stretching) 916 and 988 cm^−1^ (CC vibration) (CO stretching, CC stretching and vibration) and 1008 cm^−1^ (CO–H bending vibration)^35^ were the combinations of starch and glycerol.

After the preparation of PVDF nanofibers, the gradual addition of TPS in 5% interval figures the appearance of new peaks as shown in two insets, Fig. 3B,C. Significant new characteristic bands were observed between 3100 and 3500 cm^−1^ and the disappearance of weak peak at 2901 cm^−1^ in Pn and forming a weak broadband of 2882 cm^−1^ for 5TPn, 2872 cm^−1^ for 10TPn, 2876 cm^−1^ for 15TPn, and 2879 cm^−1^ for 20TPn. Since both polymers exhibiting thermoplastic behavior, the reactive sites of TPS in oxygen molecules readily react with the reactive sites of PVDF with hydrogen forming hydrogen bonding. Consequently, as the % of TPS increases by 5% in every sample, the hydrogen bonding increases indirect proportional since starch is a stereo-regular polymer with chirality and a high density of hydrogen bonding^36^.

Figure 3D proposes the molecular interaction between TPS and PVDF induces the formation of orientated β crystals indicates possible confirmation of hydrogen bonding at O–H^…^F–C due to the stronger polarity of the hydroxyl groups as observed in O–H stretching vibration in the range of 3500–3150 cm^−1^^37^. In addition, as investigated by Mi et al., the dipoles of β crystals relating to the peaks 1401, 1274 and 840 cm^−1^ are in the orthogonal direction and corresponding relative intensity can be used to determine the lattice orientation^38^. Meanwhile the interaction between C–H^…^F–C is a dipolar dispersion force, wherein PVDF, a non-conjugated linear fluorinated hydrocarbons and TPS also contain carbon and hydrogen resulting in transient dipole moment lead a separate transient dipole moment in neighboring molecule, initiating a fleeting attraction between the C–H molecules^39^. The remaining characteristic bands were mostly contributed by PVDF which as follows: (1) 3400–3427 cm^−1^: stretching vibration mode of the hydrogen-bonded O H groups of starch, (2) 1625–1629 cm^−1^: double bond interaction of C to C; (3) 1185–1190 cm^−1^ the retain stretching vibration of C–F; and the (4) 883–884 cm^−1^: confirmed the C=C bonding in bending movement.

#### XRD spectroscopy

**Figure 4:**
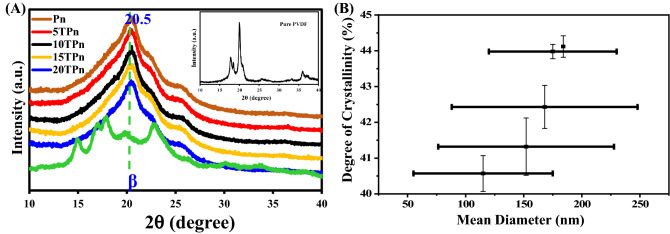
(**A**) XRD patterns and (**B**) Degree of crystallinity of PVDF, TPS, and PVDF/TPS nanofibers.

XRD is an analytical technique used to identify the crystallinity of a material; it was conducted on the TPS/PVDF nanofibers to study the effect of TPS in the PVDF fibers. Figure 4A shows the XRD patterns of the TPS/PVDF nanofibers. The PVDF patterns reveal the existence of the β-phase in the samples; the curves present a typical crystal phase peak at 20.5°. The peaks at 15°, 17.03°, 17.88°, and 22.9° correspond to the crystalline peaks of TPS. The crystallite sizes of the nanofibers are 2.28, 2.30, 2.53, 2.59, 2.87, and 1.03 nm for Pn, 5TPn, 10TPn, 15TPn, 20TPn, and TPS, respectively, as tabulated in Table 1. The calculated crystalline diameters of the PVDF nanofibers are less than that of TPS/PVDF nanofibers. The crystallite diameter indicates the level of crystallinity in a material; therefore, the crystallinity increases with the increase in the crystallite diameter as shown in Fig. 4B. The degrees of crystallinity of Pn, 5TPn, 10TPn, 15TPn, 20TPn, and TPS were 40.57, 41.32, 42.43, 43.98, 44.12, and 9.84% respectively, as summarized in Table 1. The order of the degrees of crystallinity of the nanofibers is as follows: Pn > 5TPn > 10TPn > 15TPn > 20TPn. This increment in the crystallinity indicates that the crystallinity of the PVDF nanofibers increases with the increase in the TPS content^40^.

**Table 1:** TGA, tensile, XRD, and water absorption data of TPS/PVDF nanofibers.

Name of the specimen	TGA results			Tensile test results			XRD results		Water absorbtion
	T_onset_ 10% (°C)	T_max_ 65% (°C)	Residue strength (MPa)	Tensile strength (MPa)	Tensile modulus (MPa)	Elongation at break (%)	Crystallite diameter (nm)	Degree of crystallinity (%)	
Pn	470	560	29	2.33 ± 0.12	36.85 ± 3.4	64 ± 3.08	2.28	40.57	10.88 ± 2.3
5TPn	460	500	26.5	2.78 ± 0.2	54.75 ± 5.3	53 ± 1.1	2.3	41.32	38.60 ± 1.89
10TPn	460	500	26	3.29 ± 0.17	70.9 ± 6.13	47 ± 3.2	2.53	42.43	74.86 ± 3.1
15TPn	460	500	25.2	3.3 ± 0.09	78.1 ± 3.45	42 ± 2.7	2.59	43.98	89.55 ± 4.05
20TPn	455	500	25	0.81 ± 0.1	21.08 ± 5.01	40 ± 2.04	2.87	44.12	106.58 ± 2.57
TPS	100	350	6	–	–	–	1.03	9.84	–

### Properties of TPS/PVDF nanofiber mats

#### Tensile properties

**Figure 5:**
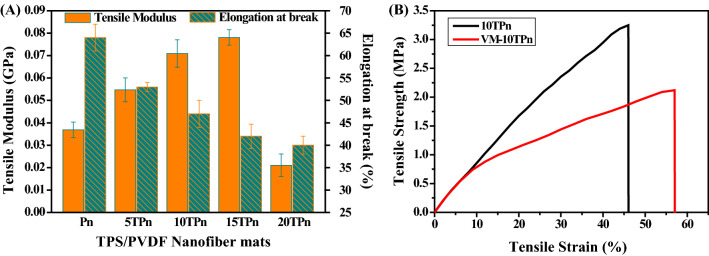
(**A**) Tensile modulus and elongation at break (**B**) stress–strain curves of TPS/PVDF nanofibers.

Tensile tests were performed on the TPS/PVDF nanofibers to determine the change in the properties after the addition of TPS in PVDF. Table 1 lists the tensile strength, modulus, and elongation at the break of the TPS/PVDF nanofiber mats. The percentages of elongation at the break of Pn, 5TPn, 10TPn, 15TPn and 20TPn are 64, 53, 47, 42, and 40%, respectively; it can also be observed from Fig. 5A that the order of the elongation at break is as follows: Pn > 5TPn > 10TPn > 15TPn > 20TPn. The elongation at the break is observed to be maximized for Pn, following which it gradually decreases with the increase in the TPS content. The elongation at break substantially decreases, indicating that the nanofiber mat becomes relatively brittle on the addition of TPS to PVDF. This might be due to the crystalline nature of TPS, which also increases the crystallinity of the TPS/PVDF nanofibers, as explained and confirmed by the XRD results. The crystalline nature of TPS changes the crystalline diameter and the percentage of crystalline in PVDF, these changes in the structure of PVDF tends to increase the brittleness in TPS/PVDF nanofibers. This nature of the brittleness of the TPS/PVDF nanofibers is highly useful for their self-healing properties when they are reinforced in composites. As the brittleness increases, the nanofibers become easily ruptured inside the composites, when damage occurs, and thus, causes the healing agent inside to easily spill out. It also helps in repairing (curing) the cracked locations.

The tensile moduli of Pn, 5TPn, 10TPn, 15TPn, and 20TPn are 0.036, 0.054, 0.070, 0.078, and 0.02 GPa, respectively, as tabulated in Table 1. Interestingly, the order of the modulus is the reverse of that of the elongation at break and is as follows: Pn < 5TPn < 10TPn < 15TPn. Only the modulus of 20TPn is very less compared to that of Pn, as shown in Fig. 5A, and the tensile moduli of 5, 10, and 15TPn are increased by 32, 48, and 52%, respectively. This change in the trend of the moduli also explains the increase in the stiffness of the fibers due to the increase in the brittleness of the TPS/PVDF nanofibers owing to the addition of TPS. As the TPS content increases, the value of the tensile modulus increases, indicating a decrement in the flexibility of the PVDF nanofibers.

The tensile strength of the TPS/PVDF nanofibers presents the same trend as the modulus. The strength of the TPS/PVDF nanofibers increases as the percentage of TPS increases by up to 15%, and subsequently, it reduces at 20% TPS. Compared to Pn, the tensile strength of 5, 10, and 15TPn is increased by 16, 29, and 29% respectively. All the fiber mats were tested after being conditioned in a laboratory environment (23 °C and 35% relative humidity) for 24 h under identical conditions. However, as the TPS content is further increased, both the tensile stress and modulus are decreased. The tensile strength and modulus of 20TPn are much lower compared to those of Pn. As the weight percentage of TPS in the nanofibers is further increased (20%), it agglomerates, which subsequently decreases the strength and modulus of the nanofibers. Moreover, the agglomeration of TPS increases the beads in the nanofibers, and thus, decreases the strength of the fibers. However, these agglomeration problems are negligible at low weight percentages.

Samples 10TPn and VM–10TPn underwent tensile tests, and the stress–strain curves are as shown in Fig. 5B. Their thicknesses were 0.12 mm and 0.26 mm, respectively, which suggests that the VM–10TPN sample is 2.16 times thicker than the 10TPn sample. This is because two additional components (VE resin combined with an accelerator and MEKP hardener) were simultaneously supplied with the TPS/PVDF solution during the co-electrospinning process. Young’s moduli E of the 10TPn mat and the VE–10TPn specimens were 0.066 and 0.041 GPa, respectively. This indicates that the 10TPn mat is fully solidified and is stiffer than the VM–10TPn mat containing the liquid resin monomer or cure in the fiber as the core. The 10TPn is a solid structure without any voids and VM–10TPn acts as a hollow structure with a liquid inside and is less in strength and modulus than 10TPn due to the differences in their structures. The ultimate tensile strength of the 10TPn mat is also almost twice that of the VM–10TPn mat, as depicted in Fig. 5B.

#### Thermal analysis of core materials

**Figure 6:**
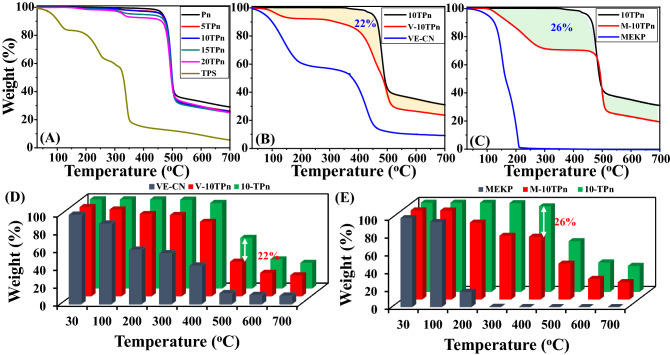
TGA curves of (**A**) TPS/PVDF, (**B**) V–10TPn, (**C**) M–10TPn and (**D**,**E**) Schematic description about volume % of healing agent (VE–CN and MEKP) in TPS/PVDF nano fibers in the light of thermal properties.

TGA is a technique that measures the thermal stability of a given specimen and is conducted on TPS/PVDF nanofibers. Figure 6A shows the thermographs of the TPS/PVDF nanofibers. Additionally, Table 1 lists the temperatures at which the specimens are reduced by 10% weight due to the evaporation of their moisture content. The total decomposition (Tmax) of Pn fibers is at 560 °C, whereas the decomposition of all TPn (5TPn, 10TPn, 15TPn, and 20TPn) fibers are at 500 °C due to the less thermal stability of TPS (Tmax 350 °C). From Fig. 5, it can be observed that the major weight loss of all the nanofibers is between 480 and 520 °C, whereas, for TPS, it is between 150 and 350 °C owing to its high combustibility, TPS presents a two-step thermal oxidative degradation at 220 and 340 °C, and a similar degradation can be observed for 5, 10, 15, and 20TPn confirming the presence of TPS in the nanofibers. The residues of Pn, 5TPn, 10TPn, 15TPn, 20TPn, and TPS are 29, 26.5, 26, 25.2, 25, and 6%, respectively, as tabulated in Table 1. This suggests that adding TPS reduces the residual PVDF nanofibers.

TGA explains the thermal degradation of the nanofibers, based on which the amount of the core material in the core–shell nanofibers can also be assessed. V–10TPn and M–10TPn were tested along with three control samples, 10TPn, and VE resin combined with CN and MEKP. Figure 6B shows the TGA plots of VE, 10TPn, and VE–10TPn. In the case of VE resin, the shape of the TGA curve in an inert atmosphere possesses two distinct mass loss steps. The first occurs at approximately 100 °C, and the second stage of the degradation occurs between 390 and 440 °C. The V–10TPn curve presents the thermal degradations of both the VE resin and 10-TPn, confirming the presence of the VE resin in V–10TPn. The VE resin contents in the core–shell nanofibers are estimated by considering the weight loss contribution from V–10TPn near 420 °C, using the weight loss of 10TPn as the baseline. Thus, the VE resin content in V–10TPn was determined to be approximately 22 wt%. Similarly, Fig. 6C shows the TGA curves of MEKP, 10TPn, and M–10TPn. The MEKP curve exhibits a one-step weight loss starting at 190 °C and continuing up to 210 °C. In the case of M–10TPn, the thermal degradation pattern shows a two-step decomposition that is a combination of both the MEKP and 10–TPn curves. Considering the same approximation, the MEKP content in M–10TPn is estimated to be approximately 26 wt%^41^. The clearest demonstration of the quantity of the core materials (VE–CN and MEKP) in V–10TPn and M–10TPn has depicted via a 3D column graph in Fig. 6D,E.

#### Water absorption test

Water absorption tests were conducted on the TPS/PVDF nanofibers to evaluate the effect of TPS on water absorption, and the results are tabulated in Table 1. The average water absorption percentages of Pn, 5TPn, 10TPn, 15TPn, and 20TPn are 10.88, 38.6, 74.86, 89.55, and 106.58%, respectively. The order of the water absorption of the fibers is as follows: 20TPn > 15TPn > 10TPn > 5TPn > Pn. The water absorption percentage increases with the increase in the TPS content in the nanofibers. The water absorption rate of PVDF is less (10.88%) than that of the TPS/PVDF fibers owing to its hydrophobic nature. Concurrently, the hydrophilic nature of TPS plays an important role in the absorption of water. Overall, the blended fibers exhibit significantly different natures than PVDF, confirming the chemical bonding of TPS with PVDF (FTIR section) and forming new types of nanofibers that are completely different from PVDF nanofibers.

### Self-healing properties

#### Confirmation of healing agent in core–shell nanofibers by FESEM

**Figure 7:**
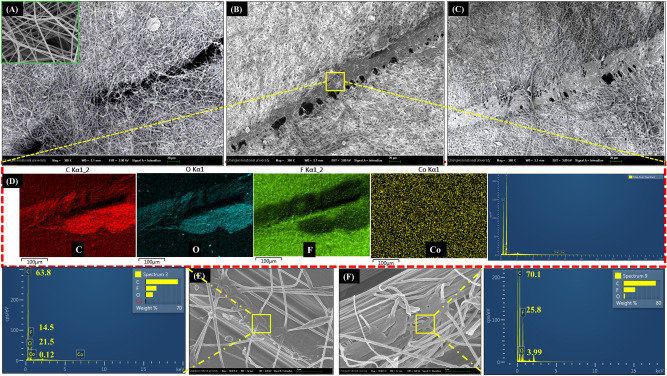
SEM images of VM–10TPn (**A**) scratched, (**B**) after 6 h, (**C**) after 24 h, (**D**) EDX mapping of VM-10TPn (**E**) ruptured V–10TPn nanofibers and EDX analysis at ruptured portion, and (**F**) ruptured M–10TP nanofibers and EDX analysis at ruptured portion.

A scratch test was performed on a VM–10TPn mat to understand the healing phenomena of the above self-healing nanofibers. Figure 7 shows the process mechanism of releasing a healing medicine from the self-healing nanofibers. A zoomed portion of Fig. 7A displays the self-healing nanofibers, in which grooves can be observed clearly at the scratched portion along with the breakage of nanofibers. Subsequently, i.e., after 6 h, as can be seen in Fig. 7B, the groove portion becomes narrow and the flooding out of the healing resin from the self-healing nanofibers at the broken parts can be observed clearly. Partial curing of the resin can be also observed in the figure, referring to the simultaneous release of the healing resin (VE–CN) along with the catalyst (MEKP). This phenomenon continued further and the healing resin filled the remaining groove portions, as depicted in Fig. 7C. For further confirmation of core healing compounds along with base shell polymer could be examined at groove portion by EDS elemental mapping as shown in Fig. 7D exhibiting the major elements i.e., carbon (C), Oxygen (O), Fluorine (F), Cobalt (Co) present in VE–CN, MEKP and 10TPn which are strong evidential for the liquid state of the core material until the curing occur. The core–shell nanofibers with VE–CN (V–10TPn) and MEKP (M–10TPn) were separately spun on carbon fibers, as illustrated in Fig. 7E,F, to display the leakage of the core material from the core–shell nanofibers. Figure 7E shows clearly the leakage of VE–CN from V–10TPn at the ruptured part and further supporting the EDS analysis showing the respective elements [PVDF (C, O, F), VE (C, O) and CN (C, Co)]. Similarly, a leakage of MEKP from the M–10TPn fibers at the ruptured portions can be seen clearly in Fig. 7F with EDX analysis exhibiting the elements of PVDF (C, O, F) and MEKP (C, O) at the ruptured portions. Overall, the fabricated self-healing nanofibers can carry sufficient amounts of the resin and are competitive with the existing nanofibers for self-healing applications^42^.

#### Self-healing phenomena during tensile testing

**Figure 8:**
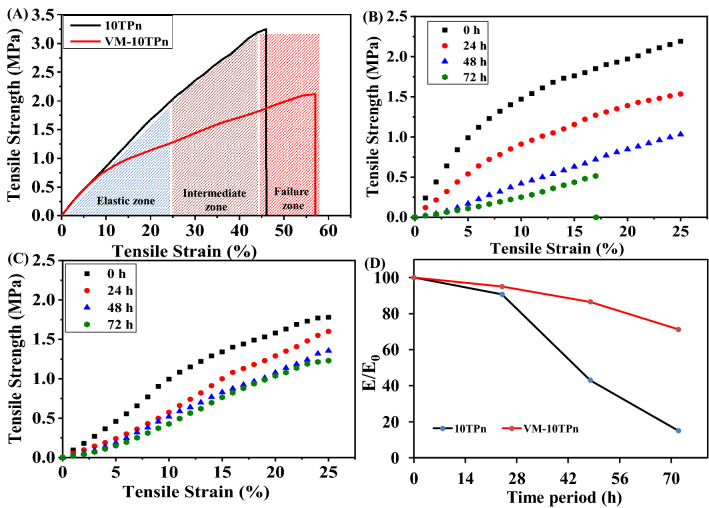
(**A**) Tensile test results, (**B**,**C**) Tensile stress/strain curves at periodic stretching, and (**D**) Relative variation in tensile modulus of 10TPn and VM–10TPn.

Figure 8 presents the healing phenomena of the self-healing nanofibers based on mechanical, digital, and morphological validations. Three deformation zones can be seen in the stress–strain curves (Fig. 8A) of 10TPn and VM–10TPN, which are categorized into elastic, intermediate plastic, and ultimately catastrophic failure zones. Till up to 25% of the strain range, the curve is linear and is in the elastic zone; the intermediate plastic zone is between the strain of 25% and 45%. Subsequently, the curve tends to rupture, which indicates the ultimate catastrophic failure zone. These deformation zones are different for different nanofiber mats (Elastic zone, plastic zone, failure zone)^43^.

Tensile tests (stretching) are performed on 10TPn and VM–10TPn periodically in time intervals of 24 h (0 h, 24 h, 48 h, and 72 h) and with a 25% strain range; the corresponding results are shown in Fig. 8B,C. The tensile strength of 10TPn at the first, second, third, and fourth stretching are 2.13, 1.58, 1.04, and 0.51 MPa, respectively. As the number of stretching increases, the strength decreases by 76%. The decrement in the strength of 10TPn is due to the breakage of a few nanofibers at each loading time; eventually, this effect induces the fiber mat losses, in terms of the original strength. Similarly, the tensile strength of VM–10TPn at the first, second, third, and fourth stretching is 1.81, 1.64, 1.4, and 1.25 MPa, respectively. Interestingly, a significant decrement in the strength is not observed owing to the self-healing phenomena. The tensile moduli of 10TPn are 66.4, 60.2, 28.59, and 10.15 MPa at the first, second, third, and fourth stretching, respectively. The moduli present similar behavior as the tensile strength. The modulus of VM–10TPn at the four periodic stretching modes are 36.38, 34.56, 31.5, and 26.8 MPa, respectively. In the initial stretching modes, the modulus abruptly decreases; however, in subsequent cases, this change is not noted. The probable reason is that in the first stretching, the breakage of the number of fibers decreases, and at each stage, curing occurs. This might suppress the stretching of the nanofibers. The flexibility of the nanofibers is comparatively higher than those of the cured portions in the fiber mat.

**Figure 9:**
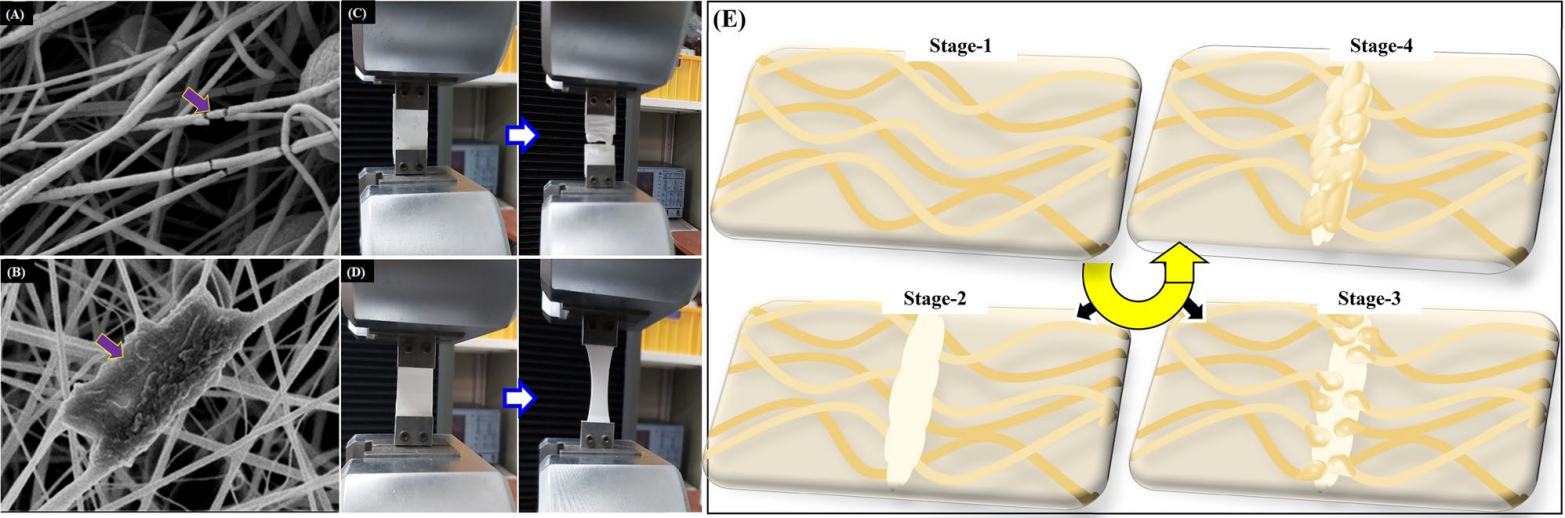
(**A**,**B**) Micro images of core–shell nano fibers, (**C**,**D**) digital images of 10TPn and VM–10TPn following repeated stretching by tensile loading and (**E**) Schematic representation of healing mechanism.

Figure 9A,B display the micro observations related to the periodical tensile test to further validate the self-healing mechanism. Figure 9A shows the broken fibers immediately after the first stretching in the direction of the applied tensile load. Some undisturbed fibers are present in the stretched mat, which will be involved in further tensile stretching. In addition, during each stretching, both the resin (VE–CN) and catalyst (MEKP)-carrying nanofibers simultaneously break. Hence, there may be a possible healing process using a curing resin, as proven in Fig. 9B. As shown in the figure, a cured resin covers a certain area where the resin flows spreads, and broken fibers can also be observed.

The self-healing mechanism is further confirmed by plotting a graph of the variations in the tensile modulus (E/E_0_) with the number of stretching for 10TPn and VM–10TPn, as shown in Fig. 8D. The modulus of the variation (E/E_0_) is calculated by considering the modulus of the first stretching as numerator (E) and the modulus of each case as the denominator (E_0_). The modulus variations of 10TPn and VM–10TPn are reduced by 85% and 29%, respectively. Both the plots apparently significantly vary owing to the nature of the fabricated nanofiber polymer and the spill out of the healing polymer. As explained in the Introduction section, PVDF is a thermoplastic polymer that is elastic in nature (elongation at the break, 64% (Table 1)), and the vinyl ester resin is a thermoset fragile polymer. Hence, in the case of VM–10TPn, the modulus slightly decreases at each stretching and finally reaches 71% at the fourth stretching because of the brittle nature of the healing resin, which also supports the increase in the modulus at each stage. Furthermore, no breakage is observed (Fig. 9D). However, in 10TPn, there is no healing concept; instead, there is an abrupt decrement in the modulus, which reaches 15%, with the eventual breakage of the nanofibers (Fig. 9C). The schematic representation is showed in Fig. 9E connected to the self-healing mechanism for physical understanding. The core–shell nano fibers (VM–10TPn) experienced in the healing process through different stages by manual damage and could be observed the healed damage portion by the process of releasing the healing resin along with curing agents simultaneously. In the schematic representation having total 4 stages as follows: Stage 1 showed the well-prepared core–shell nanofibers (VM–10TPn). The manual damaged nanofibers shown in stage-2. The core material releasing from broken nano-fibers has demonstrated in stage-3 and in stage-4 the effective bonding of nano-fiber with the chemical reaction between VE, CN and MEKP and a solid patch forming at broken edges of nano-fibers.

## Conclusions

Self-healing nanofibers are successfully prepared. The outcomes of the study are as follows: FESEM and XRD analysis supporting the mechanical properties by increasing the diameter (115 → 184 nm) and crystallinity (40.5 → 44.1%) of the PVDF nanofibers with TPS. Spectral analysis proved the formation of the green PVDF compound by chemical interaction. The TGA results of V–10TPn and M–10TPn confirmed the presence and also the quantity of VE–CN (22%) and MEKP (26%) in the core–shell nanofibers. Periodic tensile loadings within the elastic zone on the nanofibers and core–shell nanofibers showed marginal differences in the modulus. They evidentially confirmed the self-healing behavior by observing the recovery of the modulus in each stretching of the core–shell nanofibers. Moreover, microanalysis further confirmed clearly the flow of the healing resin (VE resin) at the scratched portion in the pierced fibers, and curing process was conducted automatically by interacting with the curing agents after some time. Overall, this self-healing finding may have the capability to apply the real-time structural implementation in aerospace, automobile, electronic, bio-medicinal, etc., especially for recovery of micro damages in natural/synthetic fiber-reinforced composites, the authors’ continuous future study. Moreover, these findings strongly support the research on self-healing for its further advancement and create a unique path in the direction of biodegradability, which is a major global issue in the present engineering sectors.

## Abbreviations

Pn: PVDF nanofibers
5TPn: 5% TPS/PVDF nanofibers
10TPn: 10% TPS/PVDF nanofibers
15TPn: 15% TPS/PVDF nanofibers
20TPn: 20% TPS/PVDF nanofibers
V–10TPn: VE–CN infused core–shell TPS/PVDF nanofibers
M–10TPn: MEKP infused core–shell TPS/PVDF nanofibers
VM–10TPn: Self-healing nanofibers (combination of both V–10TPn and M–10TPn)
